# NMR secondary structure and interactions of recombinant human MOZART1 protein, a component of the gamma‐tubulin complex

**DOI:** 10.1002/pro.3282

**Published:** 2017-09-27

**Authors:** Cyprian D. Cukier, Audrey Tourdes, Dounia El‐Mazouni, Valérie Guillet, Julian Nomme, Lionel Mourey, Alain Milon, Andreas Merdes, Virginie Gervais

**Affiliations:** ^1^ Institut de Pharmacologie et Biologie Structurale, IPBS, Université de Toulouse, CNRS, UPS Toulouse France; ^2^ Centre de Biologie du Développement Université de Toulouse, CNRS, UPS Toulouse France

**Keywords:** MOZART1, GCP3, NMR, oligomeric state, γ‐tubulin

## Abstract

Mitotic‐spindle organizing protein associated with a ring of γ‐tubulin 1 (MOZART1) is an 8.5 kDa protein linked to regulation of γ‐tubulin ring complexes (γTuRCs), which are involved in nucleation of microtubules. Despite its small size, MOZART1 represents a challenging target for detailed characterization *in vitro*. We described herein a protocol for efficient production of recombinant human MOZART1 in *Escherichia coli* and assessed the properties of the purified protein using a combination of size exclusion chromatography coupled with multiangle light scattering (SEC‐MALS), dynamic light scattering (DLS), and nuclear magnetic resonance (NMR) experiments. MOZART1 forms heterogeneous oligomers in solution. We identified optimal detergent and buffer conditions for recording well resolved NMR experiments allowing nearly full protein assignment and identification of three distinct alpha‐helical structured regions. Finally, using NMR, we showed that MOZART1 interacts with the N‐terminus (residues 1–250) of GCP3 (γ‐tubulin complex protein 3). Our data illustrate the capacity of MOZART1 to form oligomers, promoting multiple contacts with a subset of protein partners in the context of microtubule nucleation.

**Abbreviations**
MOZART1: mitotic‐spindle organizing protein associated with a ring of γ‐tubulin 1
γTuRC: γ‐tubulin ring complex
γTuSC: γ‐tubulin small complex
GCP: γ‐tubulin complex protein
SEC‐MALS: size exclusion chromatography coupled with multi‐angle light scattering
DLS: dynamic light scattering
NMR: nuclear magnetic resonance
LB: Luria‐Bertani
IPTG: isopropyl‐β‐*D*‐1‐thiogalactopyranoside
DTT: dithiothreitol
IMAC: immobilized metal affinity chromatography
PMSF: phenylmethylsulfonyl fluoride
DDM: N‐dodecyl beta maltoside
NDSB‐195: non‐detergent sulfobetaine‐195
LDAO: N,N‐dimethyldodecan‐1‐amine oxide
SDS: sodium dodecyl sulfate
CSI: chemical shift index

## Introduction

Microtubules are a major component of the cytoskeleton in eukaryotic cells, involved in processes such as transport, cell division, and signaling.^1^ They are highly dynamic polar structures capable of rapid rearrangements in response to stimuli.^2^, ^3^, ^4^ Microtubules polymerize from heterodimers of α‐ and β‐tubulin. The linear arrangement of tubulin dimers into protofilaments creates a polarity, with β‐tubulin oriented to the so‐called plus‐end of the microtubule, and α‐tubulin oriented toward the minus‐end. Most microtubules in the cell contain 13 protofilaments, aligned laterally in a three‐start helix with a hollow core. Assembly of microtubules involves hydrolysis of GTP to GDP. *In vitro*, polymerization of microtubules from purified α/β‐tubulin is kinetically unfavorable and produces a large percentage of polymers with a variable number of protofilaments. To ensure the correct diameter and geometry, assembly of microtubules in cells is nucleated from specific sites, involving multiprotein complexes called γTuRCs (γ‐tubulin ring complexes).^1^ Depletion of γTuRC components in cells leads to strong mitotic defects, such as aberrant mitotic spindles and error‐prone segregation of chromosomes.^5^

γTuRCs are ∼2.2 MDa multiprotein complexes composed of at least six different proteins, with molecular masses ranging from ∼70 to 210 kDa and present in single or multiple copies in the complex.^1^ The overall architecture of the γTuRCs is conserved between distant organisms such as plants, yeast, and human, but in some lower organisms (e.g., *Saccharomyces cerevisiae*), γTuRCs can have a reduced number of subunits.^1^ The structural insight into γTuRC organization comes from several cryo‐electron microscopy studies^6^, ^7^, ^8^, ^9^, ^10^, ^11^, ^12^ and from the X‐ray structures of γ‐tubulin^13^, ^14^ and γ‐tubulin complex protein 4 (GCP4), a prototype of all GCP proteins.^15^ Those studies provided important information on how γTuRCs may perform their function, leading to models of γTuRCs as structural templates for microtubule nucleation, by longitudinal binding of α/β‐tubulin dimers to the surface of γ‐tubulin. However, it remains enigmatic how cells regulate microtubule nucleation spatially and temporally, as the majority of γTuRCs are found in an inactive form, soluble in the cytoplasm.^16^ Various proteins such as pericentrin, NEDD1/GCP‐WD, and CDK5RAP2 have been implicated in the regulation of γTuRCs.^17^, ^18^, ^19^ In addition, recent studies revealed that MOZART1 (human mitotic‐spindle organizing protein associated with a ring of γ‐tubulin 1) protein may equally be involved in this regulation.^20^, ^21^, ^22^, ^23^, ^24^, ^25^, ^26^, ^27^

**Figure 1:**
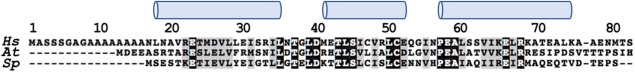
Sequence alignment of human MOZART1 protein and its plant and yeast homologs (*Hs*: *Homo sapiens MOZART1*–*NP001065243*, *At*: *Arabidopsis thaliana GIP1*–*AEE82764*, *Sp*: *Schizosaccharomyces pombe Mzt1*–*P0CF96*). The strictly conserved residues are in white on a black background and the similar residues are on a grey background. The alignment was done using Clustal Omega^44^ and rendered with Boxshade. Location of helices as determined by NMR (see below) is indicated on the top of the alignment.

MOZART1 is a relatively small protein consisting of 82 residues.^25^ Similar to other components of γTuRCs, it is conserved between different species from animals to plants and most fungi with the exception of *S. cerevisiae*, and its fission yeast and plant homologs are called Mzt1 and GIP1, respectively (Fig. 1).^20^, ^25^ These proteins are predicted to have an α‐helical fold and would interact with a 10‐amino‐acid hydrophobic sequence motif present in the N‐terminal extension of GCP3, GCP5, GCP6, and potentially of GCP2 proteins.^26^, ^27^ The binding of MOZART1 to a subset of GCPs may support the assembly of multiple γTuSCs into γTuRCs, and a cooperative binding of other proteins to the GCPs, such as proteins carrying CM1‐motifs, and NEDD1.^26^, ^27^ Furthermore, recent studies have shown that MOZART1 together with Spc110 would act as oligomerization chaperones to cooperatively promote oligomerization of γTuSCs into well‐organized and active microtubule nucleation template.^27^ Altogether, these data indicate that MOZART1 may contribute to the recruitment of γTuRCs to microtubule‐organizing centers (MTOCs), and to the activation of microtubule nucleation.^20^, ^21^, ^22^, ^23^, ^24^, ^25^, ^26^, ^27^ Consistently, defects in MOZART1 lead to abnormalities in mitotic spindle assembly and in cell division and development.^20^, ^21^, ^22^, ^24^, ^25^, ^27^

The biochemical and structural description of MOZART1 and its interaction with GCP3 and other GCPs may significantly advance our understanding of γTuRC functionality. Unfortunately, recent studies on yeast and plant homologs of MOZART1 have shown that despite its small size, Mzt1/GIP1 protein represents a challenging target for detailed characterization *in vitro*.^20^, ^25^

In this article, we report the expression and purification of recombinant human MOZART1 and the structural characterization of the purified protein using a combination of DLS, SEC‐MALS, and NMR experiments. We performed ^1^H, ^13^C, and ^15^N resonance assignments, which indicate the presence of three distinct helices. Furthermore, NMR chemical shift perturbation (CSP) analysis revealed that the three helices are involved in the interaction with GCP3. Together these data provide the first complete biochemical, biophysical, and structural characterization of human MOZART1 reported so far, paving the way for further binding studies with γTuRCs components.

## Results

### Overexpression and purification of MOZART1 protein from prokaryotic cells

The production of MOZART1 protein in *Escherichia coli* cells proved to be challenging, failing to provide any recombinant protein when the wild‐type version of the gene was used. To obtain quantities of protein required for *in vitro* characterization, we expressed the protein from a plasmid‐containing codon‐optimized version of *MOZART1* gene (Supporting Information, Fig. S1). The latter gene allowed for production of soluble MOZART1 protein in a range of temperatures (20–37°C), with the highest yield for expression at 20°C for 24 h (up to 8 mg of protein per liter of LB or minimal medium culture).

### Biophysical characterization and preparation of samples for NMR studies

**Figure 2:**
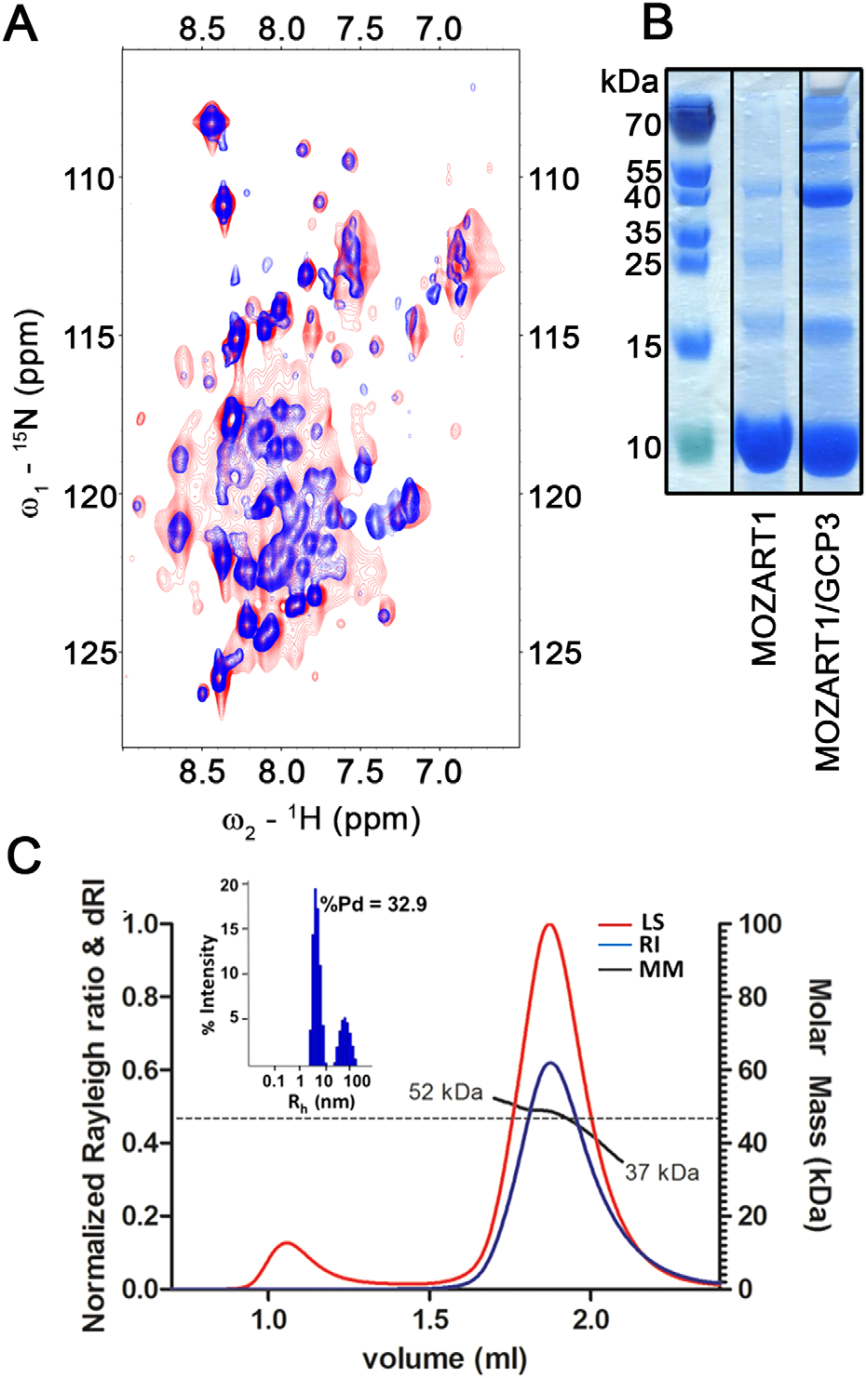
Characterization of MOZART1 protein sample. (A) ^15^N‐SOFAST‐HMQC (red) and BEST‐TROSY (blue) spectra of MOZART1 in 50 mM sodium phosphate pH 6.0, 150 mM NaCl recorded at 25°C and 600 MHz Larmor frequency. (B) Coomassie‐stained SDS‐PAGE gel of samples of the MOZART1 protein alone after IMAC and SEC chromatography (middle) and after co‐purification with the SUMO‐GCP3^(1–250)^‐His_6_ fusion protein on a HiTrap *TALON* ^®^ affinity column (*right*). (C) Elution profile examined by SEC‐MALS. The traces of light scattering (red, LS), differential refractive index (blue, RI), and MALS calculated molar mass (black, MM) are shown. The black trace indicates the MALS calculated molar mass of the eluted protein that ranges from 52 to 37 kDa. The grey dashed line indicates the average molar mass value of 46.6 kDa (the theoretical molecular weight of recombinant MOZART1 is 8.7 kDa). The histogram of size distribution obtained from DLS analysis of purified protein is shown in the window where hydrodynamic radius (*R*_h_) versus intensity percentage is represented. The major peak is centered at an *R*_h_ value of 5.05 nm. The percentage of polydispersity (%Pd) is indicated.

The initial NMR samples gave spectra of poor quality with many broad lines, making resonances unobservable, and with only a few (∼20) sharp lines [Fig. 2(A)], a disappointing result considering the low molecular mass of recombinant MOZART1 protein (8.7 kDa). Sharper lines could be obtained by employing TROSY experiments, which are optimized for measuring macromolecules of large molecular weight. Nevertheless, the number of observed signals and the quality of spectra were still not satisfactory [Fig. 2(A)]. The extensive screening of experimental conditions including pH, ionic strength, temperature, and reducing agents could only partially overcome these limitations, but not sufficiently to make resonance assignment of the protein feasible.

To better understand the difficulties encountered, we studied the oligomeric state and homogeneity of the MOZART1 protein sample. We noticed that the protein eluted at ∼60 mL on the Superdex S75 16/600 column used for purification, which corresponded to a smaller elution volume and larger size than expected for an 8.7 kDa monomer. To provide a reliable molar mass, we performed SEC‐MALS analysis,^28^ where light scattering and refractometry curves showed one elution peak giving an average molar mass of 46.6 kDa [Fig. 2(C)]. Such a value is consistent with the presence of pentameric forms. Furthermore, the molar mass range from 37 to 52 kDa raises the possibility that MOZART1 is in equilibrium between different oligomeric states (tetramer to hexamer). Notably, the presence of a smaller peak representing earlier eluted protein (0.1% of the total mass contributing to <10% of the light scattering signal) indicates a minor amount of higher molecular weight species, suggesting that MOZART1 is slightly prone to aggregation. In line with this, DLS analysis demonstrated that the MOZART1 sample was not perfectly monodispersed, revealing a polydispersity of 32.9% [Fig. 2(C)]. Altogether, these data indicate that MOZART1 does not exist in a single oligomeric state, but is rather polydisperse.

**Figure 3:**
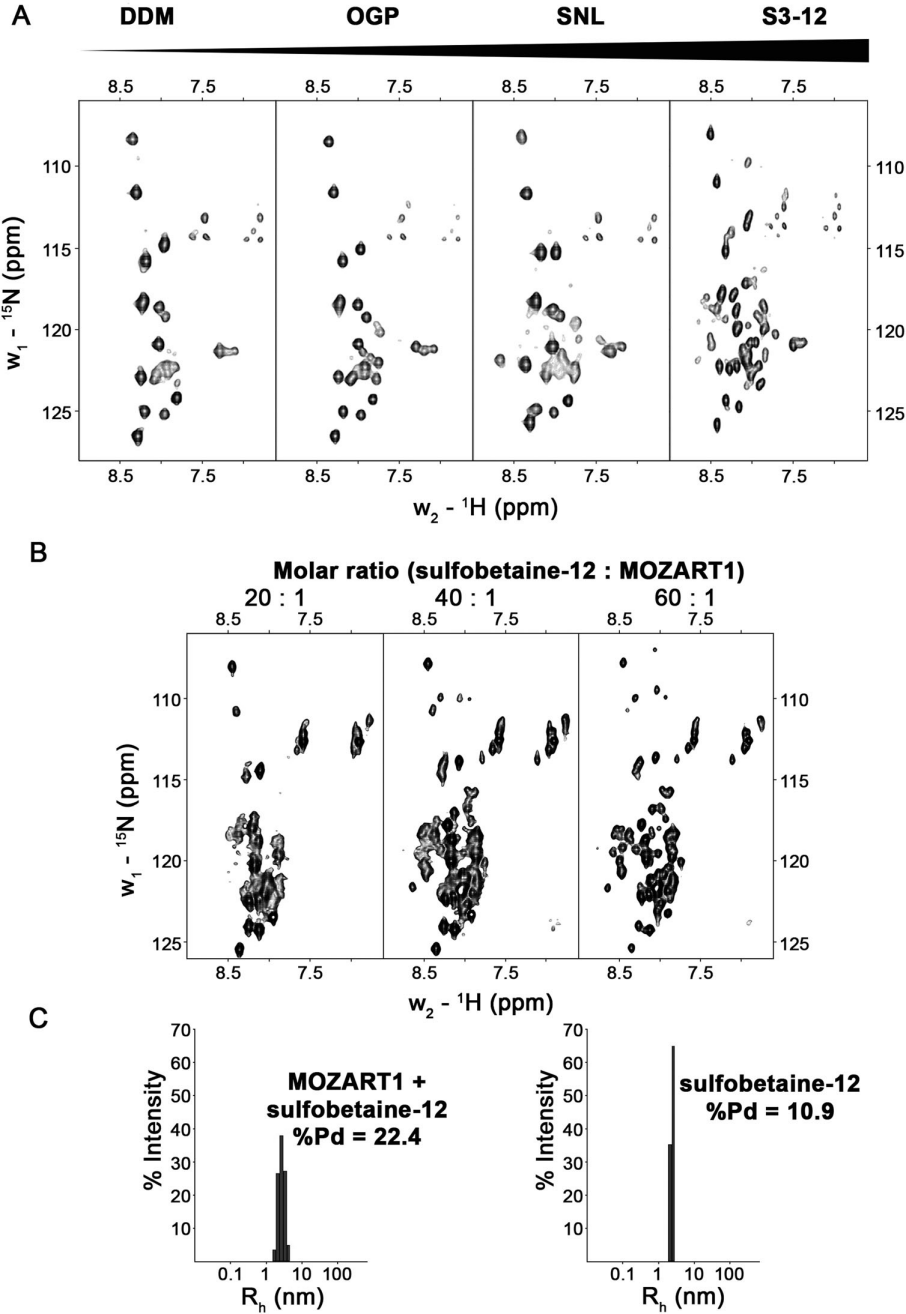
Screening of solubilizing agents on MOZART1 protein sample. (A) BEST‐TROSY spectra of MOZART1 protein in the presence of different detergents recorded at 25°C and 600 MHz Larmor frequency. The example spectra with increased improvement from left to right are presented. DDM, N‐dodecyl beta maltoside; OGP, octyl β‐*d*‐glucopyranoside; SNL, N‐lauroyl‐sarcosine; S3–12, sulfobetaine‐12. (B) SOFAST‐HMQC spectra of 150 μM MOZART1 in the presence of different concentrations of sulfobetaine‐12 detergent. The spectra were recorded at 37°C and 600 MHz Larmor frequency. (C) Histograms of size distribution obtained from DLS analysis for MOZART1 in the presence of sulfobetaine‐12 detergent (left) and for sulfobetaine‐12 detergent alone (right). %Pd, percent polydispersity.

As already mentioned, many attempts to improve the NMR spectral quality were only partially successful and NMR spectra of MOZART1, despite employing TROSY‐based experiments, were of poor quality. We then included a panel of detergent‐ and non‐detergent‐solubilizing agents into our optimization process (Table S1). The tested compounds affected MOZART1 NMR samples to several extents: from no effect (DDM, NDSB‐195) to satisfactory enhancement of NMR signals (sulfobetaine‐12, LDAO) [Fig. 3(A)]. We chose sulfobetaine‐12 to be included in NMR sample of MOZART1 protein, because it gave slightly improved NMR spectra compared with LDAO. Subsequent experiments demonstrated that sulfobetaine‐12 effect on MOZART1 spectra is concentration dependent and significant improvement is achieved at ∼60:1 molar ratio of sulfobetaine‐12:MOZART1 protein [Fig. 3(B)]. The presence of sulfobetaine‐12 indeed decreased the heterogeneity of the MOZART1 sample as revealed by the polydispersity value reduced from 32.9% to 22.4% in DLS experiments, nevertheless, still higher when compared to sulfobetaine‐12 micelles alone (10.9%) [Figs. 2(C) and 3(C)]. Hydrodynamic radii (*R*_h_) were calculated in the different conditions showing a reduction of *R*_h_ value from 5.05 nm for MOZART1 alone to 2.77 nm in sulfobetaine‐12 [Figs. 2(C) and 3(C)].

### Resonance assignment and initial structure characterization

**Figure 4:**
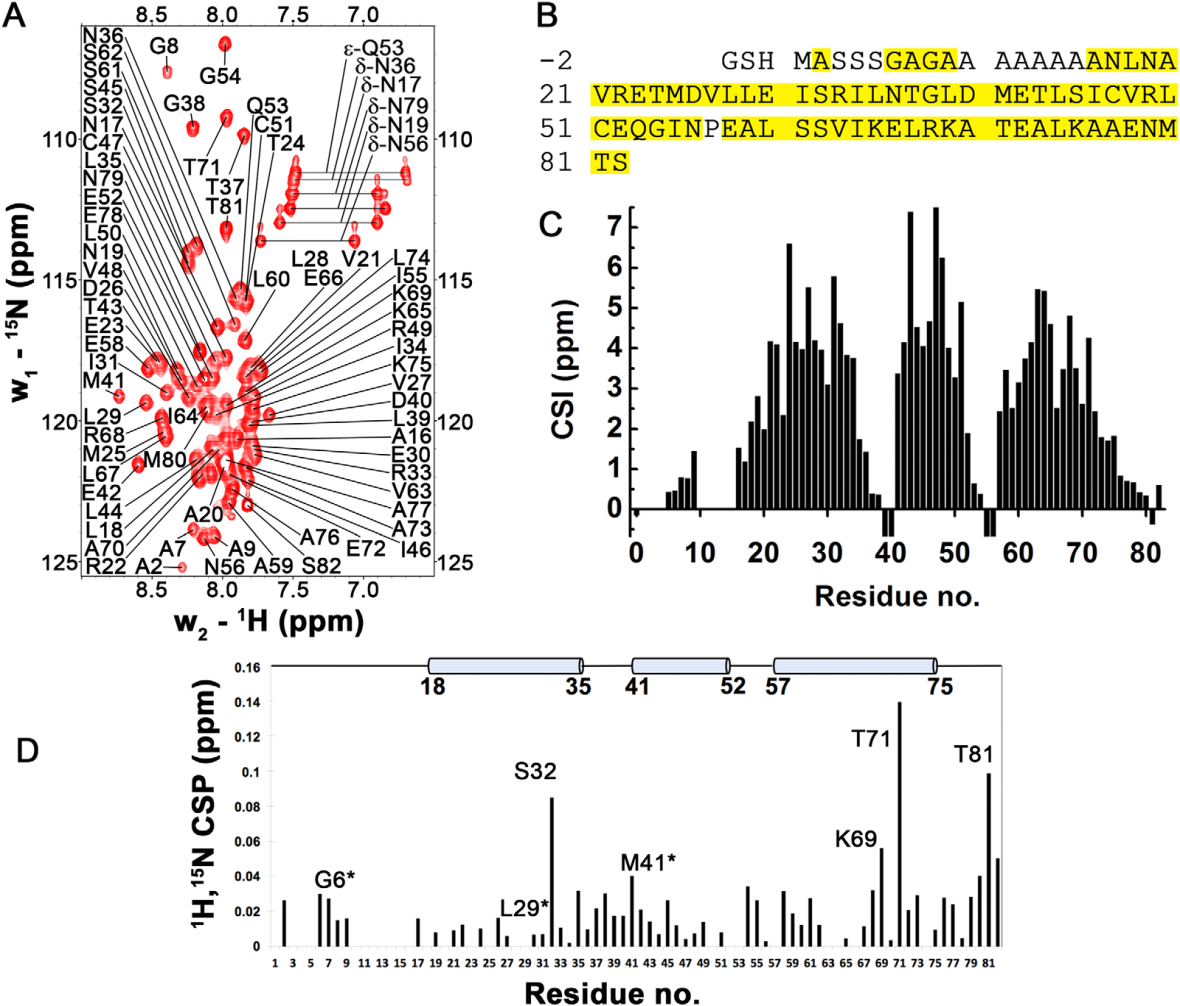
Resonance assignment and NMR secondary structure of MOZART1 protein. (A) Assigned ^15^N‐BEST‐HSQC spectrum of MOZART1 protein recorded at 37°C and 600 MHz Larmor frequency. (B) Primary sequence of MOZART1 protein with residues for which backbone amide resonances were assigned highlighted in yellow. (C) Chemical shift index (CSI = (*δ*_C_^α^_,MOZART1_ − *δ*_C_^α^_,random coil_) − (*δ*_C_^β^_,MOZART1_ − *δ*_C_^β^_,random coil_)). Random coil values were taken from Wishart et al.^45^ The positive (CSI > 1.5) values indicate clearly three well‐defined α‐helical regions in solution. (D) Chemical shift perturbation (CSP) of MOZART1 upon addition of the GCP3^(1–250)^ fusion protein. The CSPs of MOZART1 are plotted versus residue numbers with positions of helices as determined by NMR indicated on top. The residues that display chemical shift changes or whose NMR signals are significantly broadened upon titration with GCP3 are labeled. Stars indicate residues displaying signal broadening (until the limit of detection in the case of L29).

The optimized NMR conditions where the MOZART1 protein was solubilized in sulfobetaine‐12 micelles allowed us to record the 3D NMR experiments necessary for protein resonance assignment. A completeness of 86% was achieved for backbone resonances and 82% for side‐chain resonances with primarily missing assignments corresponding to residues located in the N‐terminal alanine‐rich region of the protein [Fig. 4(A,B)]. This region is not present in the homologs of MOZART1 (Fig. 1) and is most likely flexible as several sharp cross‐peaks of alanines in ^1^H–^15^N spectra could not be assigned [Fig. 4(A)]. The analysis of chemical shift values of C^α^ and C^β^ resonances demonstrated the presence of three α‐helices within MOZART1 protein (residues L18‐L35, M41‐E52, P57‐K75) [Fig. 4(C) and Supporting Information, Fig. S2). Similar boundaries were obtained from the analysis of chemical shift values of C^α^, C^β^, C′, H^N^, H^α^, and N resonances with the TALOS+ software,^29^ in which torsion angle values characteristic of α‐helix were observed for residues A16‐L35, M41‐E52, and P57‐A76. The three helices were systematically predicted by structure prediction programs such as Lomets,^30^ Phyre2,^31^ or CS‐Rosetta^32^ with comparable boundaries (Supporting Information, Fig. S2).

### NMR titration

Because MOZART1 was initially described as a GCP3‐interacting protein,^22^, ^24^ we studied the binding between these two partners in more detail. Using MOZART1 and the N‐terminal domain of GCP3 (residues 1–250), we recorded a series of SOFAST HMQC‐based chemical shift perturbation (CSP) experiments upon addition of increasing concentrations of SUMO‐GCP3^(1–250)^–His_6_ fusion protein and we followed changes in the MOZART1 chemical shifts. The chemical shift variations observed in NMR titration experiments are due to fast exchange between bound and free forms, and are best suited for weak interactions and thus low affinity complexes (typically Kd > μM). The addition of unlabeled GCP3 resulted in rather weak chemical shift changes in the HMQC spectra. Nevertheless, several residues of MOZART1 appeared to be more affected upon addition of GCP3, in terms of chemical shift changes (residues S32, K69, T71, and T81) and/or signal broadening (residues G6, L29, and M41). Most of these residues map onto the helices of MOZART1 [Fig. 4(D)].

Altogether, our NMR experiments indicate an interaction between MOZART1 and the N‐terminal domain of GCP3 on the millisecond or faster NMR exchange regime.

## Discussion

The small MOZART1 protein has recently gained more attention as an important player in the regulation of γTuRC targeting and activity.^21^, ^26^, ^27^, ^33^ In this article, we describe the protocol for expression and purification of human recombinant MOZART1 protein from *E. coli*, which allows for obtaining sufficient amount of protein for biophysical characterization *in vitro* and for structural studies. Using a combination of DLS, SEC‐MALS, and NMR, we demonstrate that MOZART1 displays self‐association properties in solution, forming several different oligomers, which are in equilibrium. The single peak observed in the SEC‐MALS experiments corresponds to a molar mass of 46.6 ± 0.1 kDa, suggesting a pentamer on average. On the other hand, DLS experiments showed that the sample is heterogeneous. Thus, heterogeneity could explain the poor quality of the NMR spectra, which is likely due to chemical exchange between the different states of MOZART1 in the sample leading to NMR line broadening. The presence of MOZART1 oligomers in solution may reflect a role of this protein in the lateral assembly of multiple γTuSCs into γTuRCs, similar to the previously documented role of Spc110 in γTuRC assembly.^26^, ^27^, ^34^, ^35^, ^36^

However, the oligomeric state renders this protein family highly challenging for *in vitro* studies. Dhani et al. showed that yeast Mzt1 protein gives three distinct peaks on a gel filtration column, possibly corresponding to a dodecamer (∼130 kDa), a heptamer/hexamer (∼50 kDa), and a tetramer/trimer (∼35 kDa).^25^ The authors also experienced poor behavior of Mzt1 protein in NMR experiments giving only about 25% of expected correlations in ^1^H–^15^N HSQC spectra.^25^ Similarly, oligomeric forms were observed in *C. albicans* (CaMzt1) and plant (GIP1) MOZART1 homologs.^20^, ^27^

We demonstrate here that the quality of MOZART1 protein sample can be significantly improved by including detergents in the sample. The best results were obtained for sulfobetaine‐12, which in molar ratio of ∼60:1 (detergent:protein) provided significant improvement of NMR signals. Interestingly, the aggregation number (number of molecules forming individual micelles) of sulfobetaine‐12 is 55–87,^37^ suggesting that there is one MOZART1 molecule per detergent micelle. Our experimental conditions provided the best quality of NMR spectra obtained so far for a MOZART1/Mzt1/GIP1 protein, allowing us to report the first nearly complete chemical shift resonance assignment for MOZART1. The protein displays three distinct α‐helical regions, adding unambiguous and accurate structural data to the predicted 3D model of AtGIP1/MOZART1.^20^ These regions largely correspond to conserved parts in all organisms.

Despite our attempts to measure at 950 MHz on a concentrated sample, we were unable to identify sufficient amount of long‐range unambiguous NOE restraints for structure calculations. Therefore, the atomic‐resolution structure of MOZART1 remains elusive.

Finally, we showed that MOZART1 is able to bind to the N‐terminal extension of GCP3 in detergent solution, and identified residues perturbed upon interaction that are located in the helices of MOZART1, thereby participating to the binding interface. In our NMR experiments, the weak binding observed between these two proteins supports the fact that MOZART1 may interact with fully assembled γTuRC rather than with individual GCP components,^26^ likely resulting in a high‐avidity interaction. Whether MOZART1 uses a unique interface to bind GCP3 and other components such as Spc110 remains to be addressed.

In summary, this study clearly establishes the tendency of MOZART1 to form oligomers in nondetergent solutions, a fact that may be linked with its capacity to promote multiple contacts with GCPs and its key role as an oligomerization chaperone for correct microtubule nucleation template assembly. In this context, our work provides experimental conditions for the observation of good‐quality NMR spectra, and will therefore contribute to future structural analysis of complexes between MOZART1 and its molecular partners, leading to a better understanding of the supramolecular organization of γTuRC complexes.

## Materials and Methods

### Cloning, protein expression, and purification

The wild‐type *MOZART1* gene was PCR amplified from a pGEX‐MOZART1 plasmid.^26^ Next, wild‐type and codon‐optimized (GenScript) versions of full‐length human *MOZART1* gene were cloned into pET28a expression vector (Novagen) introducing N‐terminal thrombin‐cleavable HisTag. Recombinant unlabeled MOZART1 protein was produced in a BL21 (DE3) strain of *E. coli* (Novagen) cultivated at 30°C in LB medium supplemented with 30 μg/mL kanamycin. Expression was induced with 0.5 mM isopropyl‐β‐*d*‐1‐thiogalactopyranoside (IPTG) (Euromedex) when the optical density at 600 nm reached 0.35–0.5. Cells were cultured for another 4 h at 30°C, harvested by centrifugation and stored at −20°C. For ^15^N‐ and ^15^N,^13^C‐labeled samples of MOZART1, LB medium was substituted with M9 minimal medium containing ^15^NH_4_Cl (Sigma) and ^13^C‐*d*‐glucose (Cambridge Isotope Laboratories) as the only source of nitrogen and carbon, respectively, and the temperature and time for expression were modified to 20°C and 24 h, respectively.

Frozen cell pellets were resuspended in equilibration buffer (50 mM sodium phosphate pH 8.0, 10 mM imidazole, 150 mM NaCl, 10% (v/v) glycerol, 1 mM dithiothreitol (DTT)) containing 0.1 mg/mL lysozyme, 0.03 mg/mL DNase I, 0.1% (v/v) Triton X‐100 and a mini‐Complete EDTA‐free protease inhibitor cocktail tablet (Roche), sonicated on ice, and centrifuged (40,000*g*, 40 min, 4°C). Recombinant MOZART1 protein was purified from the soluble fraction using immobilized metal ion affinity chromatography column (GE Healthcare Hi‐Trap™ IMAC). The bound protein was eluted using a gradient of 10–500 mM imidazole in 50 mM sodium phosphate pH 8.0, 150 mM NaCl, 10% (v/v) glycerol, and 1 mM DTT buffer. Subsequently, the fractions containing recombinant protein were pooled, concentrated to 2.5–3 mg/mL and treated with thrombin for 3 h at 20°C (Novagen, 0.9 U per 1 mg of recombinant protein) to remove the histidine tag. The thrombin was inactivated with 1 mM PMSF and the recombinant MOZART1 protein was further purified by size‐exclusion chromatography (HiLoad Superdex S75 16/600 column, GE Healthcare) using 50 mM sodium phosphate pH 8.0, 150 mM NaCl, and 2 mM DTT as a running buffer. The fractions with the recombinant MOZART1 protein were combined and the buffer was exchanged to 10 mM sodium phosphate pH 6.0, 50 mM NaCl, and 2 mM DTT by dialysis (NMR buffer). The final protein samples were concentrated to 300–400 µM, flash‐frozen in liquid nitrogen, and stored at −80°C. Protein concentration was determined using Bradford assay (Bio‐Rad). To perform NMR titration with MOZART1, the N‐terminus domain of human GCP3 (residues 1–250) was cloned into a pET14b plasmid and expressed as a SUMO‐GCP3^(1–250)^–His_6_ cleavable fusion protein. Owing to poor yield of GCP3 protein during purification preventing any NMR titration study, different strategies have been considered of which co‐purification, which resulted in improved yields. Briefly, the recombinant GCP3^(1–250)^ and MOZART1 proteins were first expressed separately, then co‐lysed and co‐purified using a HiTrap *TALON* ^®^ affinity column (GE healthcare). As the two proteins contain a histidine tag, they were first retained on the IMAC column and then separated using size‐exclusion chromatography (HiLoad Superdex S200 16/600 column, GE healthcare) [Fig. 2(B) and Supporting Information, Fig. S3]. After purification, the recombinant SUMO‐GCP3^(1–250)^–His_6_ fusion protein was concentrated to a final concentration of 5 mg/mL (190 μM) in the NMR buffer (with sulfobetaine‐12, see below) to perform NMR titration of ^15^N‐labeled MOZART1 (purified separately).

### Dynamic light scattering (DLS)

Samples of 150 µM ^15^N‐labeled MOZART1 protein in 50 mM sodium phosphate pH 6.0, 150 mM NaCl with or without 25 mM sulfobetaine‐12, and 10 mM DTT were analyzed at 20.5°C using a DynaPro NanoStar instrument (Wyatt Technology) and 4 µL disposable cuvettes (Wyatt Technology). The data were analyzed with Dynamics software (Wyatt Technology).

### Size exclusion chromatography coupled with multiangle light scattering (SEC‐MALS)

^15^N‐labeled MOZART1 protein buffered in 50 mM sodium phosphate pH 6.0, 150 mM NaCl was analyzed on a Superdex 200 Increase 5/150 GL (GE Healthcare) column with multiangle light scattering (MALS). The column was equilibrated in a 50 mM sodium phosphate 0.1 μm filtered buffer (pH 6.0 and 150 mM NaCl) on an Agilent 1260 Infinity LC chromatographic system (Agilent Technology). Data were collected using a DAWN 8+ and Optilab T‐rEX refractive index detector (Wyatt Technology). Of 150 µM protein sample, 70 µL was loaded on the column and the separation was performed at a flow rate of 0.4 mL/min at 15°C. Results were analyzed using the ASTRA 6.1 software (Wyatt Technology).

### Nuclear magnetic resonance (NMR) spectroscopy

For optimization of experimental conditions, samples of 50–350 μM ^15^N‐labeled MOZART1 protein were prepared in 90% H_2_O/10% ^2^H_2_O solutions of buffers with different pH, ionic strengths, and additives. The quality of samples was evaluated by recording various ^1^H–^15^N correlation experiments (HSQC, TROSY, BEST‐TROSY, BEST‐HSQC, and SOFAST‐HMQC) on a Bruker Avance III spectrometer equipped with TCI cryoprobe and operating at 600 MHz ^1^H frequency or on a Bruker Avance III spectrometer equipped with QCi probe and operating at 700 MHz ^1^H frequency. The range of temperature tested was 25–45°C.

For resonance assignment, ^15^N‐ or ^15^N,^13^C‐labeled MOZART1 protein samples at concentrations in the range of 0.45–0.7 mM were prepared in 95% H_2_O/5% ^2^H_2_O solutions of 10 mM sodium phosphate pH 6.0, 50 mM NaCl, 2 mM DTT, and 30–50 mM sulfobetaine‐12 (sulfobetaine‐12:MOZART1 molar ratio was always >60:1). NMR spectra were recorded at 37°C on Bruker Avance III or Bruker Avance II spectrometers equipped with TCI cryoprobes and operating at 600 or 950 MHz ^1^H frequencies, respectively. All NMR spectra were processed with Topspin 3.2 (Bruker Biospin) and analyzed using NMRFAM‐SPARKY.^38^ ^1^H^N^, ^15^N, ^13^C^α^, ^13^C^β^, and C′ resonance assignments were obtained from HNCA, BEST‐HN(CO)CACB, BEST‐HNCACB, HNCO, and HN(CA)CO experiments.^39^, ^40^ The side‐chain resonance assignments were obtained from ^15^N‐TOCSY‐HSQC (mixing time = 50 ms), (H)CCH‐TOCSY (mixing time = 11 ms), H(C)CH‐TOCSY (mixing time = 11 ms), ^15^N‐NOESY‐HSQC (mixing time = 80 or 120 ms), and ^13^C‐NOESY‐HSQC (mixing time = 80 or 120 ms).^40^, ^41^, ^42^ The assignment was deposited in BMRB under accession number 27093.

For NMR titration and interaction with the N‐terminus domain of GCP3, 2D SOFAST‐HMQC experiments^43^ of MOZART1 at the concentration of 12–20 μM were recorded at 298 K after each incremental addition of unlabeled SUMO‐GCP3^(1–250)^–His_6_. We could not use higher excess of GCP3^(1–250)^ due to degradation and/or precipitation problems. Normalized chemical shift changes of MOZART1 (CSP) were calculated as Δ*δ*_weighted_ = [(Δ*δ*_ΗΝ_)^2^ +(Δ*δ*_Ν_× 0.154)^2^]^1/2^).

## Conflict of Interest

The authors declare no conflict of interest in publishing the results of this study.

## Supporting information

Supporting Information Figure 1.

Supporting Information Figure 2.

Supporting Information Figure 3.

Supporting Information Table 1.

Supporting Information
