# Supplementary figures and images for: NMR secondary structure and interactions of recombinant human MOZART1 protein, a component of the gamma‐tubulin complex
Source: Protein Sci. 2017 Sep 27;26(11):2240–8. doi: 10.1002/pro.3282 (PMC5654863; doi:10.1002/pro.3282)

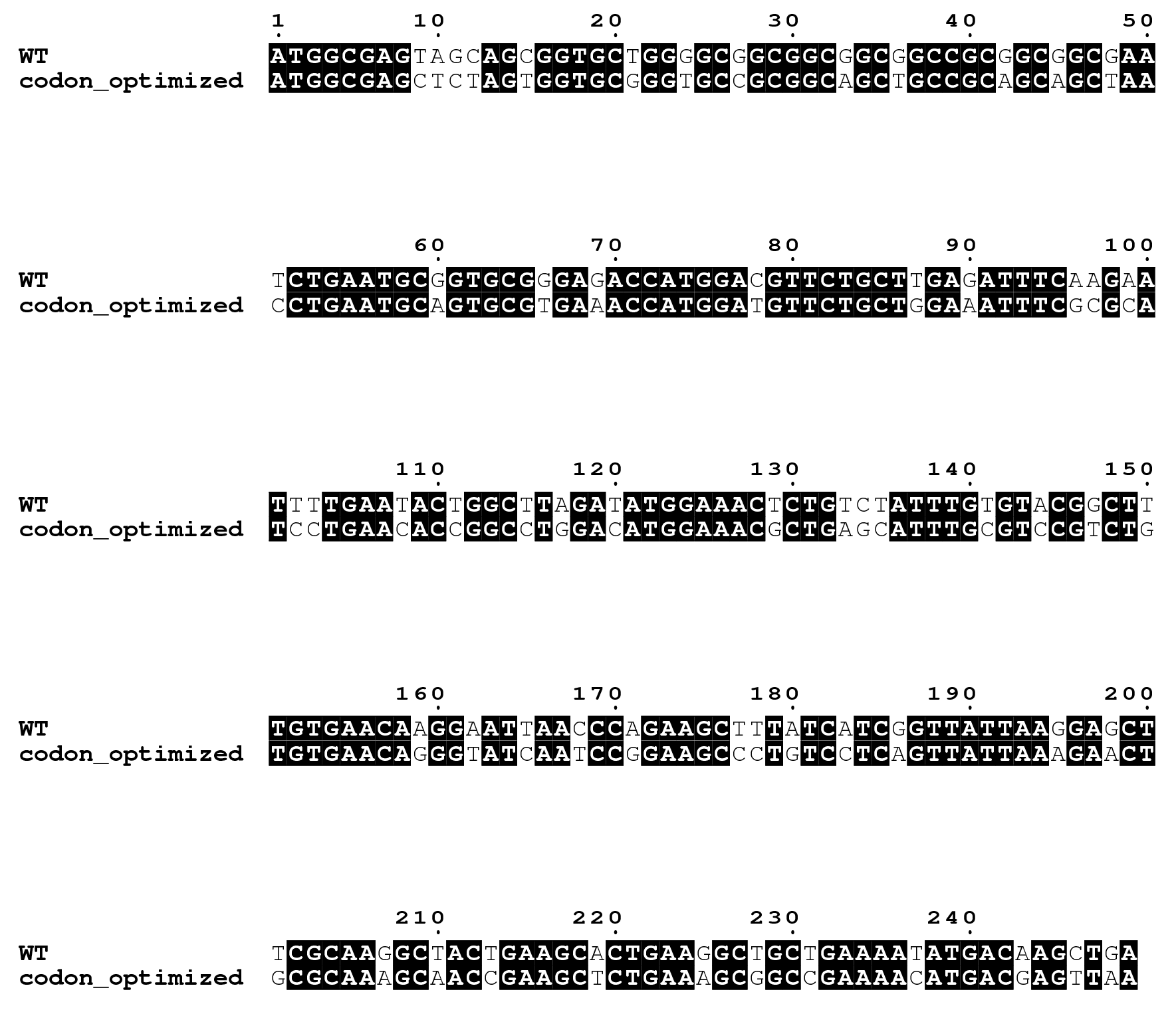

Supplement: Supplementary file 1 — Supporting Information Figure 1. [file PRO-26-2240-s001.tif]

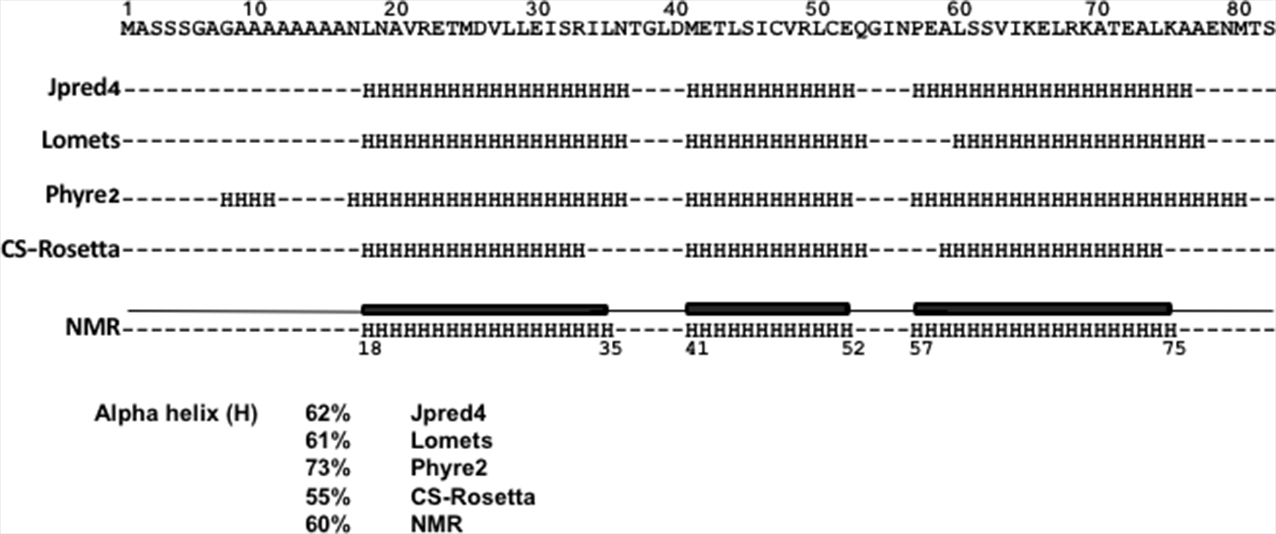

Supplement: Supplementary file 2 — Supporting Information Figure 2. [file PRO-26-2240-s002.tiff]

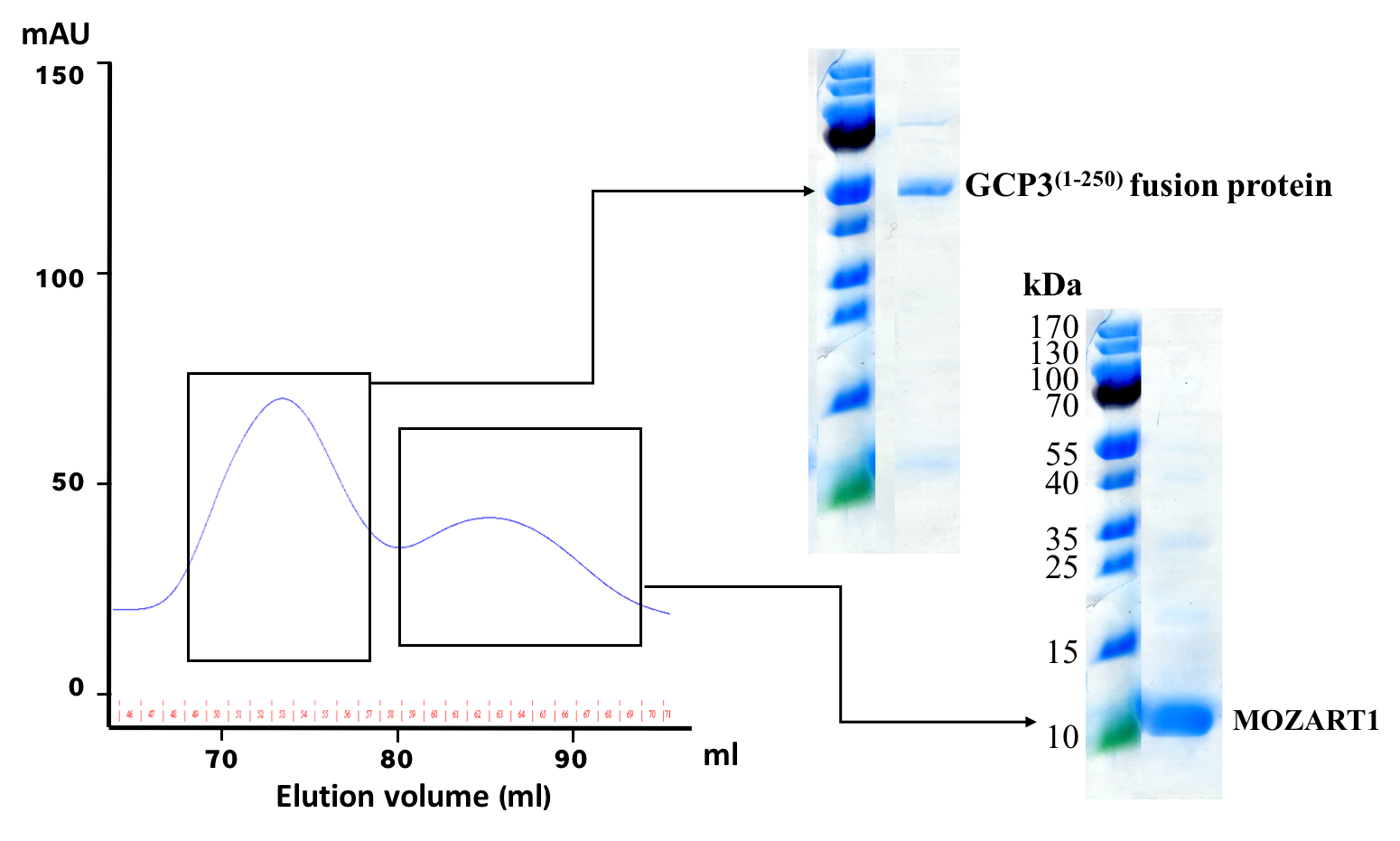

Supplement: Supplementary file 3 — Supporting Information Figure 3. [file PRO-26-2240-s003.tif]
