# Supplementary material for: NMR secondary structure and interactions of recombinant human MOZART1 protein, a component of the gamma‐tubulin complex
Source: Protein Sci. 2017 Sep 27;26(11):2240–8. doi: 10.1002/pro.3282 (PMC5654863; doi:10.1002/pro.3282)
Supplement: Supplementary file 4 — Supporting Information Table 1. [file PRO-26-2240-s004.docx]

| Reagent | IUPAC name | Charge | MW  g.mol^-1^ | Critical micelle concentration (mM)^a^ | Aggregation number^a^ | Structure |
| --- | --- | --- | --- | --- | --- | --- |
| CHAPS | 3-[dimethyl-[3-[[(4R)-4-[(3R,5S,7R,8R,9S,10S,12S,13R,14S,17R)-3,7,12-trihydroxy-10,13-dimethyl-2,3,4,5,6,7,8,9,11,12,14,15,16,17-tetradecahydro-1H-cyclopenta[a]phenanthren-17-yl]pentanoyl]amino]propyl]azaniumyl]propane-1-sulfonate | Zwitterionic | 615 | 3-10 | 4-14 | 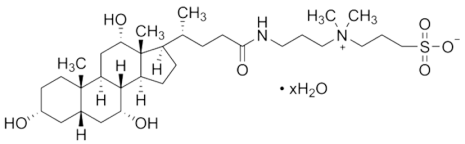 |
| NDSB-195 | 3-[ethyl(dimethyl)azaniumyl]propane-1-sulfonate | Zwitterionic | 195 | NA | NA | 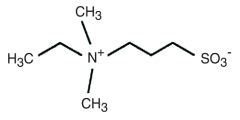 |
| Sulfobetaine-12 | 3-[dodecyl(dimethyl)azaniumyl]propane-1-sulfonate | Zwitterionic | 336 | 1.4-4 | 55-87 | 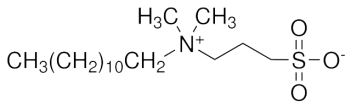 |
| LDAO | N,N-dimethyldodecan-1-amine oxide | Zwitterionic | 229 | 2.2 | 69-73 | 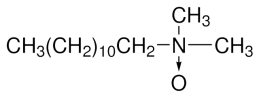 |
| Octyl β-*D*-glucopyranoside | (2R,3S,4S,5R,6R)-2-(hydroxymethyl)-6-octoxyoxane-3,4,5-triol | Non-ionic | 292 | 19-25 | 90 | 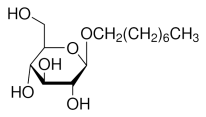 |
| N-dodecyl beta maltoside | (2R,3R,4S,5S,6R)-2-[(2R,3S,4R,5R,6R)-6-dodecoxy-4,5-dihydroxy-2-(hydroxymethyl)oxan-3-yl]oxy-6-(hydroxymethyl)oxane-3,4,5-triol | Non-ionic | 511 | 0.18 | 110-140 | 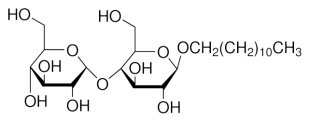 |
| N-lauroyl-sarcosine | sodium 2-[dodecanoyl(methyl)amino]acetate | Anionic | 293 | ~15 | 2 | 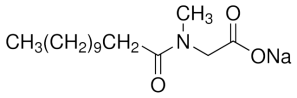 |
| SDS | sodium dodecyl sulfate | Anionic | 288 | 1.2-7.1 | 62-101 | 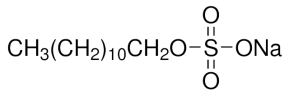 |

^a^ Critical micelle concentration and aggregation numbers are taken from le Maire *et al.* [36] and Sanders *et al.* [46]
