# Supplementary material for: NMR secondary structure and interactions of recombinant human MOZART1 protein, a component of the gamma‐tubulin complex
Source: Protein Sci. 2017 Sep 27;26(11):2240–8. doi: 10.1002/pro.3282 (PMC5654863; doi:10.1002/pro.3282)
Supplement: Supplementary file 5 — Supporting Information [file PRO-26-2240-s005.docx]

**Supplementary Material**

**Figure S1.** Sequence alignment of wild-type and codon-optimized genes encoding human MOZART1 protein. The identical residues are highlighted in black. The alignment was done using Clustal Omega [1] and rendered with ESPript 3.0 [2].

**Figure S2.** Localization of the three helices as determined by NMR (analysis of C^α^ and C^β^ chemical shift values), and comparison with various prediction programs (Jpred4 [3], Lomets [4], Phyre2 [5] and CS-Rosetta [6]). The percentage of predicted α-helix is given.

**Figure S3.** Co-purification of recombinant MOZART1 and SUMO-GCP3^(1-250)^-His_6_ fusion proteins using size-exclusion chromatography (HiLoad Superdex S200 16/600 column, GE healthcare). Closed view on the elution profile obtained for both proteins and corresponding SDS-PAGE characterization. Elution fractions are marked in red.

**Table S1.** Overview of solubilizing agents tested.

1. Sievers, F. & Higgins, D. G. (2014) Clustal Omega, accurate alignment of very large numbers of sequences, *Methods Mol Biol.* **1079**, 105-16.

2. Robert, X. & Gouet, P. (2014) Deciphering key features in protein structures with the new ENDscript server, *Nucleic Acids Res.* **42**, W320-W324.

3. Drozdetskiy, A., Cole, C., Procter, J. & Barton, G. J. (2015) JPred4: a protein secondary structure prediction server, *Nucleic Acids Res.* **43**, W389-94.

4. Wu, S. T. & Zhang, Y. (2007) LOMETS: A local meta-threading-server for protein structure prediction, *Nucleic Acids Res.* **35**, 3375-3382.

5. Kelley, L. A., Mezulis, S., Yates, C. M., Wass, M. N. & Sternberg, M. J. E. (2015) The Phyre2 web portal for protein modeling, prediction and analysis, *Nat Protoc.* **10**, 845-858.

6. Shen, Y., Lange, O., Delaglio, F., Rossi, P., Aramini, J. M., Liu, G., Eletsky, A., Wu, Y., Singarapu, K. K., Lemak, A., Ignatchenko, A., Arrowsmith, C. H., Szyperski, T., Montelione, G. T., Baker, D. & Bax, A. (2008) Consistent blind protein structure generation from NMR chemical shift data, *Proc Natl Acad Sci U S A.* **105**, 4685-90.
